# Relationship Between the Online Health Information Search and Vaccination Behavior During the COVID-19 Pandemic

**DOI:** 10.3389/ijph.2024.1606850

**Published:** 2024-11-07

**Authors:** Yunpeng Xu, Chen Pan, Peiyu Kong, Lina Shangguan

**Affiliations:** ^1^ School of Public Policy and Management, GuangXi University, Nanning, China; ^2^ School of Political Science and Public Administration, Wuhan University, Wuhan, China; ^3^ School of Marxism, Fujian Normal University, Fuzhou, China; ^4^ Department of Chemical Engineering, Imperial College London, London, United Kingdom

**Keywords:** online health information search, satisfaction with the healthcare system, vaccine risk perception, perceived usefulness of information, vaccination behavior

## Abstract

**Objectives:**

During the COVID-19 pandemic, online health information search has been shown to influence the public’s health beliefs, risk attitudes, and vaccination behavior. This study constructs a conditional process model to explore how online health information search impacts public vaccination behavior, considering critical factors like healthcare system satisfaction, vaccine risk perception, and the perceived usefulness of information.

**Methods:**

Data from the 2021 Chinese General Social Survey (N = 2,547) were analysed. The study utilized logistic regression, path analysis, and the Bootstrap method to test the conditional process model.

**Results:**

Increased online health information search promotes vaccination behavior, while increased vaccine risk perception hinders vaccination behavior. Higher satisfaction with the healthcare system encourages vaccination behavior, but online health information search reduces healthcare system satisfaction. Satisfaction with the healthcare system and vaccine risk perception play a chain mediating role between online health information search and vaccination behavior. Additionally, the perceived usefulness of information has a negative moderating effect on online health information search and healthcare system satisfaction.

**Conclusion:**

The research findings provide new insights for health information dissemination and vaccination interventions.

## Introduction

From 30 January 2020, when WHO declared the COVID-19 pandemic a “public health emergency of international concern” to 4 May 2023, when WHO declared “the end of the global health emergency caused by COVID-19 pandemic,” the global tally has exceeded 760 million confirmed cases, resulting in over 6.9 million deaths [1], posing severe challenges to the global public health governance. During the pandemic, encouraging and mobilizing the public to receive COVID-19 vaccines has been widely recognized as the safest and most effective measure for controlling the spread of the disease in China. Vaccination boosts the immune system, lowers the risk of infection, reduces the severity of cases, helps achieve herd immunity, and mitigates the overall impact of the pandemic [2, 3]. However, due to public’s limited knowledge about vaccine and exposure to negative information, vaccine hesitancy, skepticism, and opposition have arisen, making vaccination efforts more challenging [4]. To enhance their knowledge about the pandemic and vaccines, the public typically seeks information through various channels, which then influences their decision on whether to adopt health protective behaviors [5].

However, due to pandemic restrictions, the public in China heavily relies on online health information search to gain relevant knowledge, shape their perceptions and emotions towards the pandemic and vaccines, and ultimately guide their behavior [6]. Although online health information search offers rapid access to abundant information facilitating health decisions, it also poses challenges such as information overload, fragmentation, and verifiability issues. Coupled with the public’s preference for negative information, this tendency frequently results in negative risk perception and erroneous behavioral responses [7]. Therefore, how online health information searches influence public vaccination behavior is the focal point of this study.

Vaccination is not just a behavioral choice; it is a complex process involving changes in attitude, cognition, and behavior. The Knowledge-Attitude-Practice (KAP) model suggests that individual behavior change is influenced by their knowledge and attitude. It divides public behavior change into three phases: acquiring knowledge, changing attitudes, and transforming behaviors, where knowledge is the foundation, attitude is the key, and the ultimate goal is behavioral change [8]. Knowledge and attitudes towards COVID-19 play a decisive role in shaping public adherence to healthcare policies [9]. Public knowledge is largely influenced by the pandemic information they have access to. People who search for health information more frequently tend to acquire more information about the pandemic and health-related knowledge [10]. It can be observed that the frequency of online health information searches is positively correlated with public knowledge growth. Therefore, online health information searches can influence vaccination behavior by shaping public attitudes.

Since the Knowledge-Attitude-Practice (KAP) model is derived from the Theory of Reasoned Action, it places greater emphasis on cognitive factors in the process of attitude change, often overlooking the role of emotional factors in decision-making and attitude shifts. However, emotions and feelings directly impact individuals’ perceptions of risk and decision-making processes, often playing a pivotal role in shaping risk-related decisions [11]. Therefore, this study incorporates the Dual-System theory of decision-making into the model to construct a more comprehensive analytical framework, addressing the limitations of the KAP model. The Dual-System theory of decision-making suggests that in the process of attitude change and decision-making, both the heuristic and analytic systems play goal is behavioral change [12]. The analytic system shapes cognitive attitudes, while the heuristic system shapes emotional attitudes, which together form the two key dimensions of public attitude [13].

During the pandemic, the public actively seeks information about COVID-19 and vaccines to enhance their knowledge and learn preventive measures. However, due to the complexity and uncertainty of pandemic and vaccine-related information, it is crucial for professional healthcare institutions to convey crisis information and manage the situation. As a result, public satisfaction with the healthcare system becomes a key factor in heuristic judgments, influencing their emotional attitudes [14]. Generally, heuristic decision-making—where individuals rely on past experiences (such as unquestioningly following government directives)—tends to lead to correct outcomes [15]. Nevertheless, to achieve optimal decisions and a sense of self-efficacy, the public’s analytic system assesses the risks, shaping their risk perceptions. Consequently, in addition to following recommendations from healthcare systems, individuals rationally evaluate the vaccine information they gather, adjusting their cognitive attitudes toward vaccines and ultimately deciding whether to receive the vaccination [16]. Therefore, this study integrates the Knowledge-Attitude-Practice (KAP) model with the Dual-System theory of decision-making to construct a comprehensive analytical framework. This framework aims to clarify how public attitudes, such as satisfaction with the healthcare system and vaccine risk perception, influence the relationship between online health information searches and vaccination behavior.

The Dual-System Theory of decision-making posits that motivation and ability differentiate public decision-making processes, especially when considering complex or risk-related behaviors such as health choices [17]. During online health information search, only the information perceived as useful will influence the public’s health attitudes and behaviors [18]. Individuals with different information processing abilities and motivations will perceive the usefulness of information in varied way, which in turn affects how they adopt that information within the dual-system of decision-making. Therefore, perceived usefulness of information is incorporated into the research model as a moderating variable.

Overall, this study mainly contributes in the following aspects: Firstly, this study focuses on vaccine risk perception rather than the commonly used COVID-19 risk perception, which complements previous research. Secondly, this study focuses on actual vaccination behavior rather than vaccination intention, which has been the focus of most previous studies. Additionally, the data used in this research were collected during the COVID-19 pandemic, reducing recall bias and enhancing the authenticity and reliability of the findings. Lastly, and most importantly, this study integrates the Knowledge-Attitude-Practice (KAP) model with the Dual-System Theory of decision-making to construct a research model that analyzes the relationship and mechanisms between online health information searches and public vaccination behavior in the context of the COVID-19 pandemic. This aims to offer valuable insights for future governance of public health crises.

### Research Hypothesis

Health information search broadly defined as the public’s access to information about their own health, health risks, diseases, and health promotion, is often considered a key step in adopting healthy lifestyles and preventive behaviors [19]. The information that the public obtains through online searching can influence their risk perception and preventive behaviors [20]. During the COVID-19 pandemic, faced with stringent lockdown measures, the public heavily relies on the internet to access pandemic information and acquire preventive knowledge. However, as the frequency of online health information search increases, the probability that the public will be exposed to unknown and negative information environment increases, which can lead to anxiety and fear among the public, affecting the public’s pandemic risk perception and vaccination behavior [21]. In addition, the convergence of online “infodemic” and public online health information searches can exacerbate negative emotions among the public, subsequently diminishing their willingness to get vaccinated [22]. A study on HPV vaccines indicates that as the public encounters an increasing amount of vaccine information, it paradoxically reduces their willingness to receive vaccinations [23]. Based on this, the study exploratively proposes the following hypothesis:

H1 Online health information search will significantly and positively influence public vaccination behavior.

Vaccine risk perception can be summarized as an individual’s perception of negative effects after vaccination. The quantity and nature of vaccine information constitute pivotal determinants affecting the public’s vaccine risk perception and willingness to vaccinate [24]. When the public receives a substantial volume of negative vaccine-related information, they tend to question the safety of the vaccine and develop a heightened level of vaccine risk perception [25]. Consequently, as the frequency of public health information searches increases, so does their perception of vaccine risks [26]. The Protection Motivation Theory suggests that individuals’ assessments of vaccine threats and pandemic response can alter their cognition, thereby influencing their protective behaviors in response to health threats [27]. Concerns regarding vaccine safety and a heightened perception of risk are primary obstacles to vaccine uptake [28]. Based on this, the study proposes the following hypothesis:

H2 Vaccine risk perception plays a negative mediating role in the relationship between online health information search and public vaccination behavior.

Online health information search and information overload not only affect the public’s knowledge of vaccines, but also the public’s emotions [29]. To address information overload, personalized recommendation algorithms based on public preferences are extensively employed on the internet. However, this also leads the public to continuously access one-sided and homogeneous information during their health information searches, trapping them in an “information echo chamber,” which in turn impacts their satisfaction and trust in the healthcare system [30]. For instance, the more attention they pay to negative information about the pandemic, the more similar content is recommended by smart algorithms, diminishing their satisfaction with the healthcare system [31]. During the COVID-19 pandemic, healthcare institutions serve as both disseminators of pandemic information and advocates for COVID-19 vaccines. The majority of the public rely more on the healthcare system to make heuristic decision. If they do not trust the healthcare system and healthcare workers, it is difficult for them to generate positive protective behaviors [22]. Public trust and satisfaction with healthcare agencies can effectively reduce susceptibility to misinformation and promote vaccination [23]. Based on this, the study proposes the following hypothesis:

H3 Satisfaction with the healthcare system plays a negative mediating role in the relationship between online health information search and public vaccination behavior.

Furthermore, public emotions can influence public risk perception and risk decision-making through psychological shortcuts [32]. For instance, individuals closely associated with healthcare departments exhibit higher levels of risk perception and are more likely to engage in proactive protective behaviors [33] The public’s satisfaction with the healthcare system serves not only as a heuristic decision-making pathway but also shapes their risk perception [34]. Based on this, the study proposes the following hypothesis:

H4 Vaccine risk perception and satisfaction with the healthcare system play a chain mediating role in the relationship between online health information search and public vaccination behavior.

The perceived usefulness of information is a value judgment that refers to an individual’s ability to exclude useless information and select the information that aligns with their needs to aid decision-making [35]. The public’s online health information search primarily encompasses obtaining pandemic-related information and filtering out informational noise. Individuals with varying information processing capabilities and digital health literacy incorporate different “useful information” into the dual decision-making systems, subsequently influencing their perception of vaccine risks and satisfaction with the healthcare system. Individuals with high perceived usefulness of information spend more effort searching for epidemic information and incorporate more useful information into the decision-making dual system, which improves the quality of decision-making [36]. Whereas individuals with low perceived usefulness of information tend to spend effort searching for information with little gain, leading to anxiety and unease, or even giving up utilization of information [37]. Based on this, the study proposes the following hypotheses:

H5 Perceived information usefulness positively moderates the relationship between online health information search and the dual-process system of decision-making.

H5a Perceived information usefulness positively moderates the relationship between online health information search and vaccine risk perception.

H5b Perceived information usefulness positively moderates the relationship between online health information search and satisfaction with the healthcare system.

**FIGURE 1:**
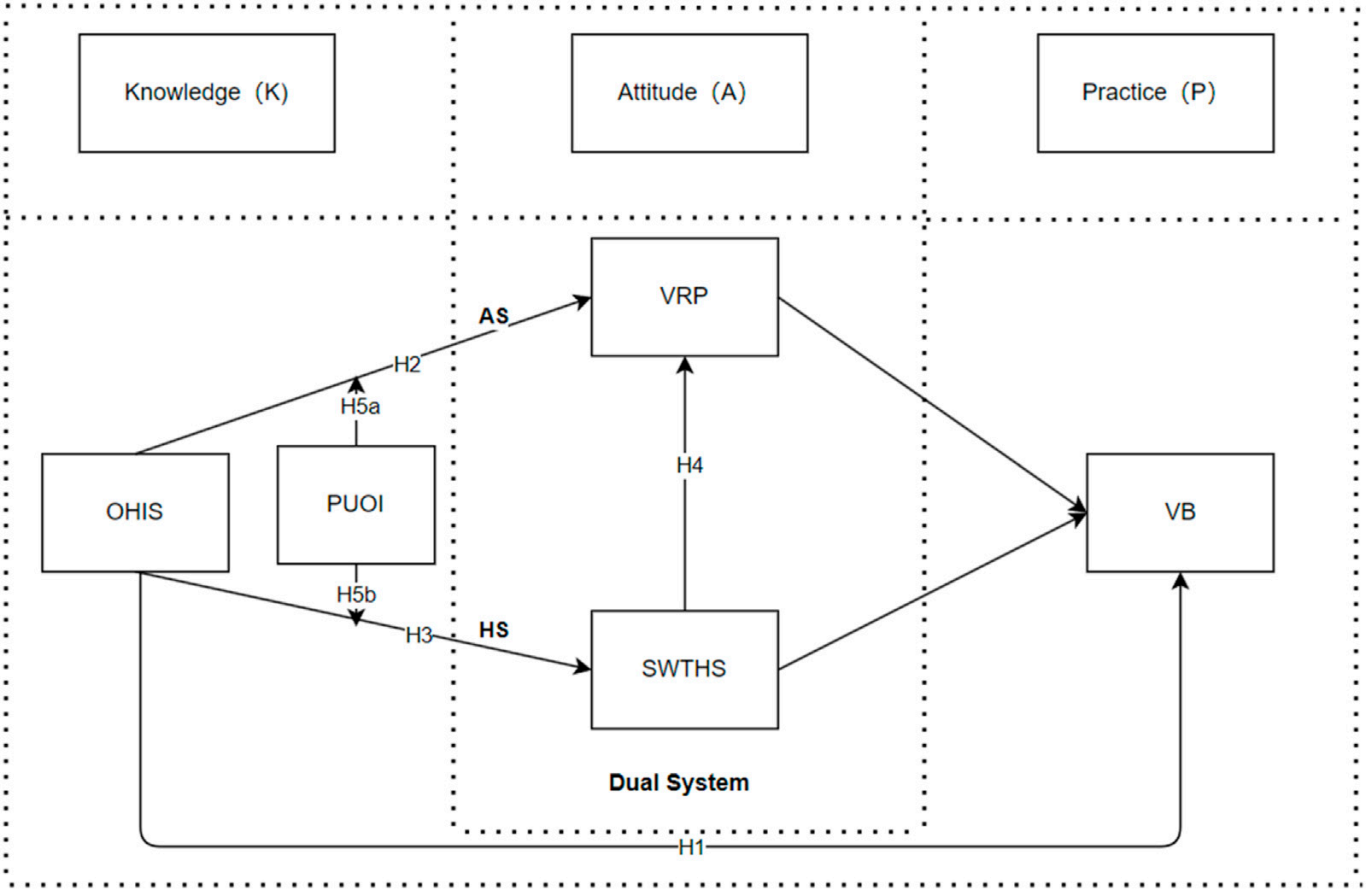
Research model (China, 2023). Note: OHIS, Online Health Information System; VRP, Vaccine Risk Perception; SWTHS, Satisfaction with The Healthcare System; VB, Vaccination Behavior; PUOI, Perceived Usefulness of Information; AS, Analytic System; HS, Heuristic System.

In summary, we propose the following model for the research (see Figure 1).

## Methods

### Data Sources

**FIGURE 2:**
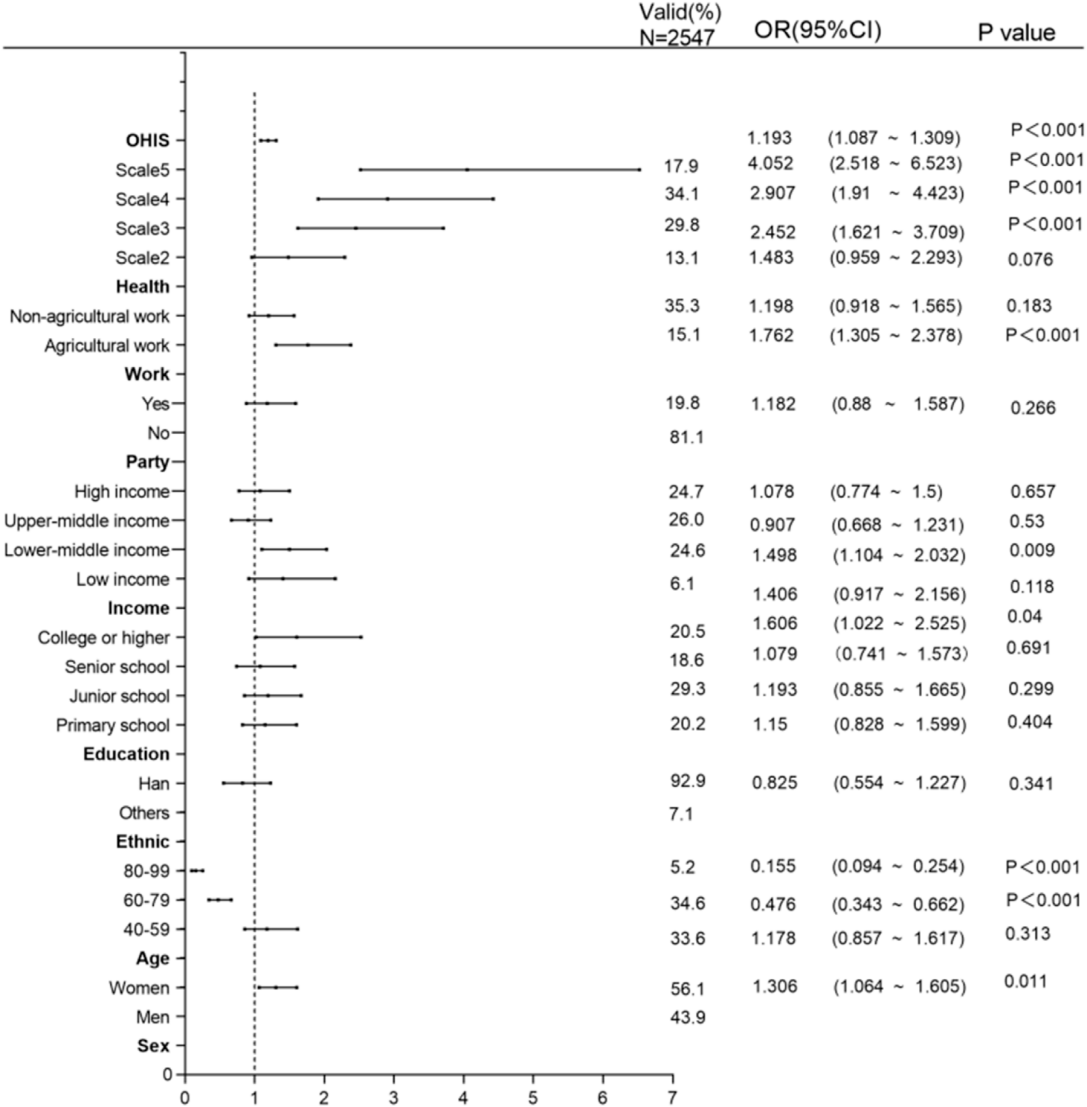
Tree diagram of binary logistic regression results (China, 2023).

The data used in this study is the 2021 Chinese General Social Survey (CGSS, publicly released on 31 March 2023). The CGSS is a large-scale, nationwide, continuous sampling survey project conducted by the Survey and Data Center of Renmin University of China using a probability sampling method and household interviews. The survey program mainly includes (a) core modules, including socio-demographic attributes, health, lifestyle, social attitudes, etc., (b) thematic modules, including the comprehensive impact of the COVID-19 pandemic, marriage and work occupation, etc., and (c) additional modules. After data screening and outlier treatment based on the research themes and related variables of this study, a final dataset of 2,547 observation samples are obtained (See Figure 2 for more detailed information).

### Measures

Measures of all variables in this study were constructed based on the items in the CGSS questionnaire.

#### Online Health Information Search Behavior

Based on the study by Xiong et al. [38], the frequency of online health information search was selected as a proxy variable for online health information search in this study. The measurement item is “In the past 12 months, how frequently did you search for health or medical information for yourself or others through various forms of internet access?”. The answer options included “never, several times a year, several times a month, several times a week, several times a day,” assigned values from 1 to 5 (M = 1.98, SD = 1.267).

#### Vaccination Behavior

The measurement item is “Currently, have you received the COVID-19 vaccine?” The responses were “yes” and “no,” assigned values of 1 and 0, respectively (M = 0.74, SD = 0.439).

#### Vaccine Risk Perception

The measurement item for vaccine risk perception is “In general, the disadvantages of vaccination outweigh the benefits.” The answer options range from “strongly disagree” to “strongly agree,” assigned values from 1 to 5. Higher scores indicate higher vaccine risk perception (M = 2.08, SD = 1.016).

#### Satisfaction With the Public Health System

The measurement item for public satisfaction with the healthcare system is “In general, are you satisfied with China’s healthcare system?” The answer options range from “very dissatisfied” to “very satisfied,” assigned values from 1 to 5. Higher scores indicate higher satisfaction (M = 4.07, SD = 0.844).

#### Perceived Usefulness of Information

This measure was adapted from Rains [39]. Perceived usefulness of information was measured using two questionnaire items, “In the past 12 months, information on the Internet has had a positive impact on my health behaviors” and “In the past 12 months, information on the Internet has helped me to understand what my doctor has told me.” “The answer options range from “strongly disagree” to “strongly agree,” assigned values from 1 to 5. Higher scores indicate a higher perception of information usefulness (M = 3.31, SD = 0.730).

#### Control Variables

The control variables included gender, age, ethnicity, education, income, party membership, nature of work, and health status.

### Analysis

**FIGURE 3:**
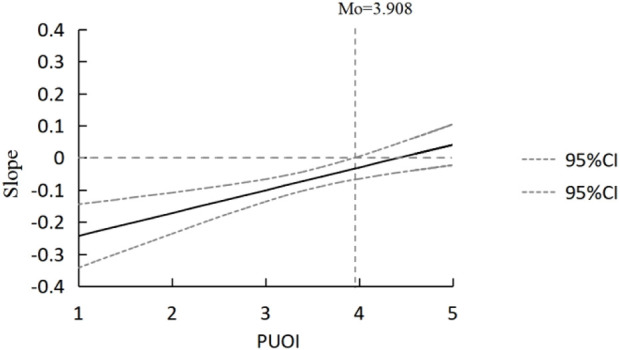
Johnson-Neyman plot (China, 2023).

To begin with, a binary logistic regression analysis was conducted to examine the impact of online health information search on vaccination behavior. The analysis results were visualized in a forest plot (see Figure 2). Next, in accordance with Hayes’ Bootstrap method, a conditional process model was examined utilizing the SPSS PRPOCESS macro. The Bootstrap method offers the advantage of assessing specific pathways while controlling for the influence of other paths when testing multiple mediating variables [40]. In this study, Model 84 in the macro was employed to test for mediation and moderation effects. The results are presented in Table 1 and Figure 3. Finally, a detailed analysis and discussion of the research findings were conducted.

**TABLE 1:** Conditional process analysis (China, 2023).

Items	Model 1	Model 2	Model 3
	SWTHS	VRP	VB
OHIS	−0.307*** (0.069)	0.057 (0.085)	0.175*** (0.047)
SWTHS		−0.082*** (0.024)	0.233*** (0.057)
VRP			−0.186*** (0.045)
PUOI	−0.089** (0.044)	0.047 (0.054)	
OHIS × PUOI	0.070*** (0.019)		
OHIS × SWTHS		−0.013 (0.024)	
Control variables	Control	Control	Control
Constant	4.410***	2.454***	0.305
*R* ^2^	0.044	0.011	N/A
F value	10.606***	2.404**	N/A

Note: ***P < 0.001, **P < 0.01, *P < 0.05.

## Results

### Main Effects Analysis Results

Based on Figure 2, incorporating the control variables into the model resulted in a *p*-value greater than 0.05 for the Hosmer and Lemeshow test, indicating a good model fit. Cox and Snell *R* ^2^ = 0.118, Nagelkerke *R* ^2^ = 0.172, the model is acceptable in social science research [41]. Online health information searching significantly positively influences vaccination (β = 0.176, *P* < 0.001, OR = 1.193), Hypothesis H1 is supported.

### Conditional Process Model Testing Results

The conditional process model testing results are presented in Table 1 and Figure 3. Model 1 shows that online health information search has a significant negative impact on public satisfaction with the healthcare system (β = −0.307, *p* < 0.001). Perceived usefulness of information has a significant negative moderating effect on the impact of online health information search on public satisfaction with the healthcare system (β = 0.070, *p* < 0.001), H5b is supported. Model 2 indicates that online health information search has no significant impact on vaccine risk perception (β = 0.057, *p* > 0.05), H2 is not supported. Perceived usefulness of information has no significant moderating effect on the impact of online health information search on vaccine risk perception (β = −0.013, *p* > 0.05), H5b is not supported. Model 3 shows that public satisfaction with the healthcare system significantly positively influences public vaccination behavior (β = 0.233, *p* < 0.001), H3 is supported. Vaccine risk perception has a significant negative impact on public vaccination behavior (β = −0.186, *p* < 0.001). Public satisfaction with the healthcare system has a significant negative impact on vaccine risk perception (β = −0.082, *p* < 0.001), H4 is supported.

**FIGURE 4:**
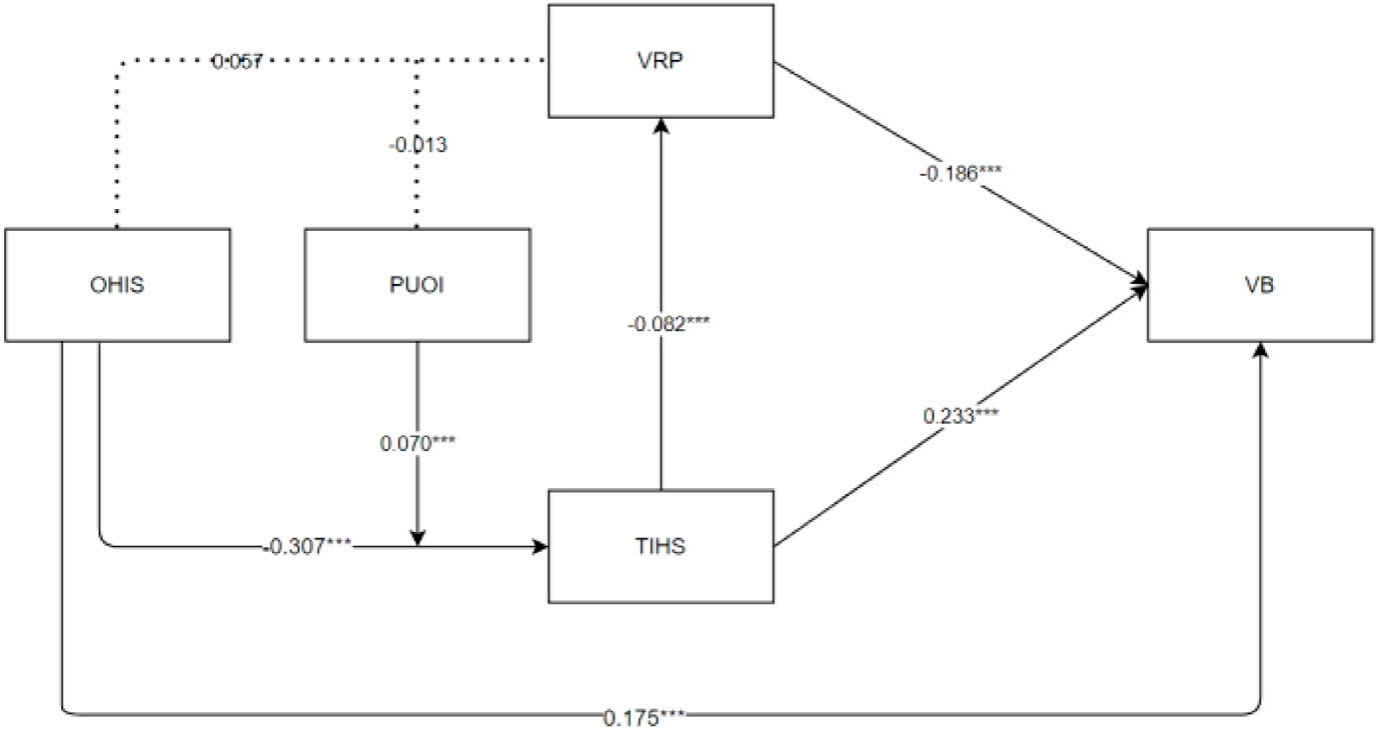
Conditional process model test results (China, 2023).

Besides, we use the Johnson-Neyman method [42] to conduct a deeper analysis, detecting the threshold point of significant and non-significant moderation effects, M0 = 3.908 (see Figure 3). When the public perceives information as very useless to useful (1–3.908 ≈ 4), an increase in perceived information usefulness significantly mitigates the negative impact of online health information search on satisfaction with the healthcare system. This suggests that within the range of 1–3.908, as the perceived usefulness of information increases, it can effectively mitigate the negative impact of online health information searches. However, when the perceived usefulness exceeds 3.908, the significant moderating effect will no longer exist. This implies that the usefulness of the information significantly influences public satisfaction with the healthcare system, particularly when engaging in online health information searches. Useless information may lead to distrust or dissatisfaction with the healthcare system, whereas useful information can have a positive effect. Finally, based on the results of the conditional process modelling tests, Figure 4 was obtained.

## Discussion

Based on the dual-system theory of decision making and the KAP model, this study focuses on exploring the relationship between the online health information search and vaccination behaviors of the Chinese citizens during the COVID-19 pandemic, including the mediating roles of vaccine risk perceptions and satisfaction with the healthcare system, as well as the moderating roles of perceived usefulness of information. This study yields the following conclusions and implications.

### Online Health Information Search and Vaccination Behavior

Online health information search during the pandemic has a significant positive effect on vaccination. This unexpected result may be attributed to variations in the urgency of information needs and the degree of government control over online information between everyday scenarios and crisis situations. While challenges like information overload and information infodemics may disrupt the public’s online health information search, leading to anxiety and fear [43], it is in times of crisis that the public’s need for a rapid acquisition of a substantial amount of information to make informed decisions becomes even more critical. During the pandemic, government authorities have intensified their focus on addressing cyberchondria and combating online rumors. In mainland China, for example, stricter regulations for managing online public discourse were implemented, with individuals who spread false information and caused significant consequences facing legal consequences. Official media outlets leveraged social platforms such as TikTok to disseminate vaccine information and promote health awareness. Hence, individuals with a higher frequency of online health information search are more likely to access accurate vaccine information and emotional support, making them more inclined to comply with government directives and get vaccinated [44]. Meanwhile, frequent online health information searches can enhance public vaccine literacy, and higher levels of vaccine literacy play a vital role in reducing vaccine hesitancy and promoting positive vaccination behaviors [45].

The result suggests that government agencies should recognize the importance of online health information search during the pandemic and formulate formal policies to regulate social media usage and dissemination of information on these platforms. It is crucial to acknowledge the dual-edged nature of new media and work towards establishing online community guidelines to curb the spread of misinformation [46]. Moreover, since one of the primary purposes of public online information search is to seek emotional support [47], government authorities should incorporate empathetic communication strategies when engaging with the public. This approach can enhance emotional connections between the government and the public, fostering proactive engagement.

### Online Health Information Search and Vaccine Risk Perception

In contrast to the findings of the Betsch et al. [25], online health information search appears to have a limited impact on vaccine risk perception. This difference can be explained by two main factors. First, vaccine risk perception is largely informed by the public’s analytical processing, which requires individuals to critically evaluate and rationally absorb vaccine related information. Merely increasing exposure to risk information does not necessarily heighten risk perception; it only does so when the information is perceived as credible and balanced [48]. Second, vaccine risk perception is a multidimensional construct, making it overly simplistic to attribute its formation solely to individual online health information searches. Instead, frequent exposure to media is more likely to influence risk perception at the collective, societal level rather than purely on an individual basis [49].

### Online Health Information Search and Satisfaction With the Healthcare System

Our study confirms that online health information search negatively impacts public satisfaction with the healthcare system. As an affective heuristic, satisfaction with the healthcare system is influenced by external depictions of health risks and individuals’ emotional experiences. In the online environment, the public often encounters conflicting and contradictory health information, leading to confusion and anxiety, which diminishes their satisfaction with the healthcare system [50]. Additionally, the fragmented nature of online information dissemination, combined with the traffic-driven algorithms of social media platforms, often shifts attention towards negative public sentiment. This focus on negativity amplifies feelings of distrust and confusion, further contributing to a decline in public satisfaction with the healthcare system [30].

Effective health information management is critical to addressing the uncertainties that arise during pandemics. Public health system reputation largely hinges on their ability to provide timely and accurate information during crises [51]. To prevent vaccine-related panics and ensure public trust, to prevent vaccine-related panics and ensure public trust, healthcare systems must proactively meet the public’s emotional and informational needs by offering reliable, transparent information before negative narratives take hold [52]. This approach is essential for maintaining public satisfaction and encouraging proactive protective behaviors.

### Vaccine Risk Perception and Satisfaction With the Healthcare System

The findings indicate that public satisfaction with the healthcare system significantly positively influences public vaccine uptake, while vaccine risk perception has a significantly negative impact on vaccine behavior. These two factors play a chain-mediated role in the relationship between online health information search and vaccine behavior. The study found that greater public trust in the healthcare system significantly reduced concerns and perceived risks about vaccines. This could be because those who trust medical institutions take the pandemic more seriously and are more likely to follow government advice, including getting vaccinated [53]. Government agencies should fully acknowledge that vaccine risks, often shaped by subjective perceptions, function as “social facts.” To address this, authorities must actively engage in disseminating accurate vaccine information and conducting educational outreach to improve public understanding and reduce the influence of perceived vaccine risks.

Our study reached a similar conclusion to a study from Switzerland, which found that higher trust in public health institutions positively influences vaccination intentions [54]. High satisfaction with the healthcare system fosters positive expectations about the competence and motivation of healthcare providers, which encourages individuals to comply with vaccination recommendations [23]. Government agencies should treat public satisfaction as a form of “social capital”—enhancing the credibility of healthcare institutions can increase public trust and compliance with health policies. Additionally, satisfaction with the healthcare system significantly reduces vaccine risk perception, underscoring the role of emotions in shaping individual risk assessments [55].

### The Role of Perceived Information Usefulness

Our study indicates that perceived usefulness of information negatively moderates the relationship between online health information search and satisfaction with the healthcare system. Individuals who perceive the information as more useful may have stronger information processing abilities or a greater belief in the healthcare system’s effectiveness, thereby mitigating the negative impact of online health information search on their overall satisfaction. For these individuals, perceived usefulness of information reflects their level of digital health literacy, which plays a crucial role in addressing challenges like information overload and infodemics [56].

To address the challenges posed by infodemics and information overload, improving public digital health literacy must be a priority. Additionally, optimizing information access channels is crucial to reduce the interference of false or unnecessary information. Healthcare systems should ensure that pandemic and vaccine-related information is systematically organized and disseminated in a structured, evidence-based manner. Especially, health authorities should use more positive message framing when sharing information, such as emphasizing the benefits of vaccination rather than the risks, to increase public willingness to get vaccinated [57]. This approach would better align health information with public needs and cognitive patterns, making it easier for people to understand and act on. Moreover, enhancing communication between healthcare providers and the public is essential to help individuals better understand health information. Strengthening these interactions can foster trust, improve public satisfaction with the healthcare system, and promote proactive engagement in protective behaviors like vaccination.

### Limitation

Our study has the following limitations. First, there are certain deficiencies in the measurement of relevant variables. Since we used secondary survey data, most variables were evaluated using a single-item question, single-item measurement may increase variance and weaken effects [39]. In certain cases, using a single-item measure to assess variables is widely accepted, particularly when the variables are relatively simple or very clear. This approach reduces the burden on respondents, ensuring efficient data collection and minimizing response fatigue [58]. Rossiter also pointed out that when the concept being measured is unidimensional and straightforward (e.g., satisfaction or specific behaviors), a single-item measure can provide accurate and valid results [59]. In this study, variables such as “online health information search” “vaccine risk perception (cost-benefit analysis)” “satisfaction with the healthcare system” and “actual vaccination behavior” are relatively simple and clear in their dimensions. Given that the data collection occurred during the outbreak of COVID-19 in China, using single-item measures was a practical decision to reduce respondent fatigue. This approach aligns with previous studies and best practices in the field. However, in future research and surveys, multiple-item measures could be considered for these variables to test the robustness and differences in the results.

Second, due to the use of CGSS (Chinese General Social Survey) data, there are limitations in the collection of certain variables. As a result, we were unable to directly include factors such as whether vaccination was mandatory, the online information environment, or the level of trust in the healthcare system prior to the pandemic in our analysis. Although these factors are likely to influence the study’s outcomes, the constraints of the data source prevented further empirical analysis. Future research should incorporate these dimensions to gain a more comprehensive understanding of public vaccination behaviors and the underlying influencing factors.

Third, this study used cross-sectional data, which can only explore the causal relationships and effects between online health information search and vaccine behavior at a specific point in time. However, the impact of public online health information search on their health attitudes and vaccination behavior is a long-term and complex integrated process. Therefore, the best way to overcome these limitations are to create surveys, conduct long-term cohort studies and obtain time-series data.

### Conclusion

Our study demonstrates that online health information search has a significant positive impact on public vaccination behavior during the COVID-19 pandemic. However, it negatively influences satisfaction with the healthcare system. Despite this, satisfaction with the healthcare system plays a crucial positive role in promoting vaccination and serves as a negative mediator between online health information search and vaccination behavior. While vaccine risk perception is not directly affected by online health information searches, it has a negative impact on vaccination behavior. Importantly, satisfaction with the healthcare system and vaccine risk perception jointly mediate the relationship between online health information search and vaccination behavior. Additionally, the perceived usefulness of information mitigates the negative effects of online health information search on healthcare satisfaction.

These findings emphasize the need for strategies that not only advocate for the importance of vaccines but also enhance the communication of health information. To improve public satisfaction and support vaccination efforts, healthcare systems must provide clear, reliable, and accessible information, addressing issues such as information overload and cyberchondria. Additionally, government agencies should work to reduce the public’s subjectively constructed risk perceptions of vaccines, thereby minimizing barriers to vaccination. Furthermore, implementing digital health literacy education is essential in helping the public identify accurate health information and mitigate the negative effects of misinformation on healthcare satisfaction. This represents a long-term strategy to combat infodemics and foster positive health behaviors.
